# Supplementary material for: Epicardial electrical heterogeneity after amiodarone treatment increases vulnerability to ventricular arrhythmias under therapeutic hypothermia
Source: PLoS One. 2023 Apr 20;18(4):e0282943. doi: 10.1371/journal.pone.0282943 (PMC10118167; doi:10.1371/journal.pone.0282943)
Supplement: S1 File — (DOCX) [file pone.0282943.s001.docx]

**Epicardial electrical heterogeneity after amiodarone increases vulnerability to ventricular arrhythmia in swine model under hypothermia**

Supplementary materials

Supplementary Table 1: Baseline data of experimental pigs

Supplementary Table 2: Electrocardiographic measurements during therapeutic hypothermia and the treatment of amiodarone

Supplementary Figure 1. The experimental protocol of therapeutic hypothermia and the treatment of amiodarone.

Supplementary Figure 2: Segmentation of the epicardium

Supplementary Figure 3. Bipolar epicardial voltage after therapeutic hypothermia or the treatment of amiodarone

Supplementary Figure 4: Activation pattern of the ventricular epicardium

Supplementary Figure 5: Western blot of the connexin 43 between control group and study group.

***Swine preparation***

Fourteen pigs (32.3 ± 4.4 kg, 26.5–40.0 kg, Supplementary Table 1) were intubated and received general anesthesia with isoflurane (concentrations of 2% for induction and 0.5% for maintenance, Baxter Healthcare Corporation, Deerfield, IL, USA) after an overnight fasting and premedication with Zoletil 50 (5 mg/kg, Virbac, Taipei, Taiwan).

***Induction and maintenance of TH***

The temperature was controlled using the Arctic Sun™ (MediVance Inc., Louisville, CO, USA) temperature-controlling system, which circulated temperature-controlled water through hydrogel energy transfer pads under negative pressure to maintain the target temperature.^1^ Using this system, we applied cooling pads to the back, chest, and thighs. The automatic mode was set to a target temperature of 33.5°, and the maximum cooling rate was used. No ice bags were used.

***Vulnerability to ventricular arrhythmias***

The ventricular arrhythmia vulnerability was determined by burst right ventricular pacing (RVP, 20 beats) to assess the vulnerability of ventricular arrhythmias. The initial pacing cycle length (PCL) of burst pacing was 400 ms, 350 ms, and 300 ms. Below the PCL of 300 ms, the PCL was decreased by 10 ms until loss of 1 to 1 ventricular capture. The burst RVP was performed in the pigs under BT, after TH, and after the infusion of amiodarone and TH, respectively. (Supplementary Figure 1) The ventricular arrhythmias were defined as sustained ventricular tachycardia (VT) or ventricular fibrillation (VF) requiring defibrillation. The burst RVP was performed to assess the vulnerability to ventricular arrhythmias in three animals that received TH and amiodarone treatment and four shams-controlled pigs under BT (36–37°C). The longest cycle length of the burst RVP enough to induce ventricular arrhythmias was defined as the threshold for the ventricular arrhythmias.

***Tissue sampling***

We obtained the tissue from the pre-specified 13 segments after sacrifice. A fixation procedure was performed immediately in both 20% formalin and liquid nitrogen to prevent sample degradation. We obtained the cardiomyocytes from both endomyocardium and epicardium.

***Immunofluorescent staining and western blotting***

Based on the resulted of the initial analysis from the CV and the change of LE durations, segment 5 (posterior basal LV), segment 8 (anterior mid RV), and segment 12 (anterior mid LV) were chosen for the connexin 43 analysis.^2^ Six sections were obtained from the above-mentioned segment. The protein expression of connexin 43 in each segment was analyzed. The samples were fixed with 4% paraformaldehyde and then permeabilized with 1% Triton X-100 (X198-07, BioShop, Burlington, Canada) for 20 min at room temperature. Images were captured using LSM700 (Carl Zeiss AG, Thornwood, NY, USA). The sections were then incubated with the appropriate primary antibody (connexin 43 polyclonal antibody, C6219, 1:100, Sigma-Aldrich, St. Louis, MO, USA) and Alexa Fluor-conjugated secondary antibodies (A-11008, 1:400, Invitrogen, Carlsbad, CA, USA). The nuclei were counterstained with an antifade reagent containing 4,6-diamidino-2-phenylindole (DAPI, Molecular Probes, D8417, 1:1000, Sigma-Aldrich).

ImageJ software was used to evaluate the intensity and lateralization of connexin 43 expressions. Assisted by image analysis software, the percentage of total tissue area occupied by connexin 43 immunoreactive signals, and the percentage of connexin 43 signal located outside the end-to-end cell junctions (lateralization). The percentages of lateralization in specific segment were means of the 24 slides (6 slides from segment 5, 8, and 12 of each pig) from the specific segment. The cardiomyocytes with well-defined cell borders were selected to measure. The intensity and lateralization of connexin 43 were quantified as those cells which were double positive for α-sarcomeric actinin and connexin 43 per 6 fields for each segment.

**Western blotting analysis**

The western blots were analyzed as previously described.^2^ Cultured cells were homogenized in RIPA lysis buffer (1×, Millipore, Billerica, MA, USA) with protease and phosphatase inhibitors (78442, Thermo Fisher Scientific). The protein homogenates were centrifuged, and protein concentrations were determined by BCA protein assays (23225, Thermo Fisher Scientific). The denatured proteins (30 µg/lane) were separated on Novex 10-20% Tricine protein gels (EC6625BOX, Invitrogen) and NuPAGE 4-12% Bis-Tris gradient gels (NP0335BOX, Invitrogen) then transferred to polyvinylidene difluoride (PVDF) membranes (Millipore). The proteins were detected by primary antibodies, including anti-Connexin 43 / GJA1 antibody (1:1000, ab87645, Abcam, Cambridge, UK) and mouse anti-GAPDH (1:1000, Thermo Fisher Scientific). The HRP-conjugated secondary Peroxidase-AffiniPure Mouse Anti-Goat IgG (1:10000, Jackson ImmunoResearch, Pennsylvania, USA) antibodies were used as the secondary antibodies. Protein expression was analyzed using AlphaEaseFC 4.0 (Alpha Innotech, San Leandro, CA, USA). Myocardial tissue from the ventricle was collected immediately after the animals were sacrificed in four pigs. Another myocardial tissue was collected immediately after the animals were sacrificed in three pigs without TH.

***Statistical analysis***

All analyses were performed using the SPSS statistical software, version 20.0. The baseline characteristics and electrophysiological parameters were reported as means ± standard deviations for continuous variables and as percentages for categorical variables. The ECG and electrophysiological parameters among the three conditions (BT, TH, and amiodarone/TH) were compared using a paired t-test. The box plot was used to showed the distribution of the parameters from the ECG and 3D electro-anatomic mapping during different condition. The horizontal line in each box is the median, and the boxes show the 10th, 25th to 75th, and 90th percentiles of the distribution of values in each group. The electrophysiological parameters between the different heart segments were compared using one-way ANOVA and post-hoc analysis with the Bonferroni method. Categorical variables were compared using the chi-square test. Statistical significance was set at P-values of < 0.05. The data underlying this article are available in the article and in its online supplementary material.

| \| **Supplementary Table 1 Baseline data of experimental pigs** \| \| \| --- \| --- \| \| Number \| 14 \| \| Body weight (kg) \| 32.3 ± 4.4 \| \| LVEF (%) \| 63.1 ± 2.2 \| \| Body temperature (°C) \| 36.9 ± 0.7 \| \| Systolic blood pressure (mm Hg) \| 101.9 ± 11.2 \| \| Heart rate (bpm) \| 102.1 ± 9.5 \|   LVEF = left ventricular ejection fraction |
| --- | --- | --- | --- | --- | --- | --- | --- | --- | --- | --- | --- | --- | --- | --- |

| \| **Supplementary Table 2: Electrocardiographic measurements during therapeutic hypothermia and the treatment of amiodarone** \| \| \| \| \| \| --- \| --- \| --- \| --- \| --- \| \|  \| BT \| TH \| Amiodarone/  TH \| p value^@^ \| \| QRSd (ms) \| 99.1 ± 2.1 \| 106.5 ± 3.1* \| 142.3 ± 4.6^#^ \| < 0.001 \| \| QT interval (ms) \| 389.4 ± 9.4 \| 473.7 ± 8.5* \| 536.7 ± 13.1^#^ \| < 0.001 \| \| Corrected QT interval (ms) \| 518.7 ± 11.9 \| 605.7 ± 14.7* \| 659.7 ± 17.3^#^ \| < 0.001 \| \| TpTn (ms) \| 48.7 ± 1.0 \| 56.8 ± 2.0* \| 86.7 ± 7.3^#^ \| < 0.001 \| \| ^@^ Repeat measurement  *: P<0.05 *P <0.05, BT vs. TH (pair-t test);  ^#^:P <0.05, TH vs. amiodarone/TH (pair-t test).  BT = baseline temperature; HT = therapeutic hypothermia; \| \| \| \| \| |
| --- | --- | --- | --- | --- | --- | --- | --- | --- | --- | --- | --- | --- | --- | --- | --- | --- | --- | --- | --- | --- | --- | --- | --- | --- | --- | --- | --- | --- | --- | --- | --- | --- | --- | --- | --- |

**Supplementary Figure 1. The experimental protocol of therapeutic hypothermia and the treatment of amiodarone.**

**
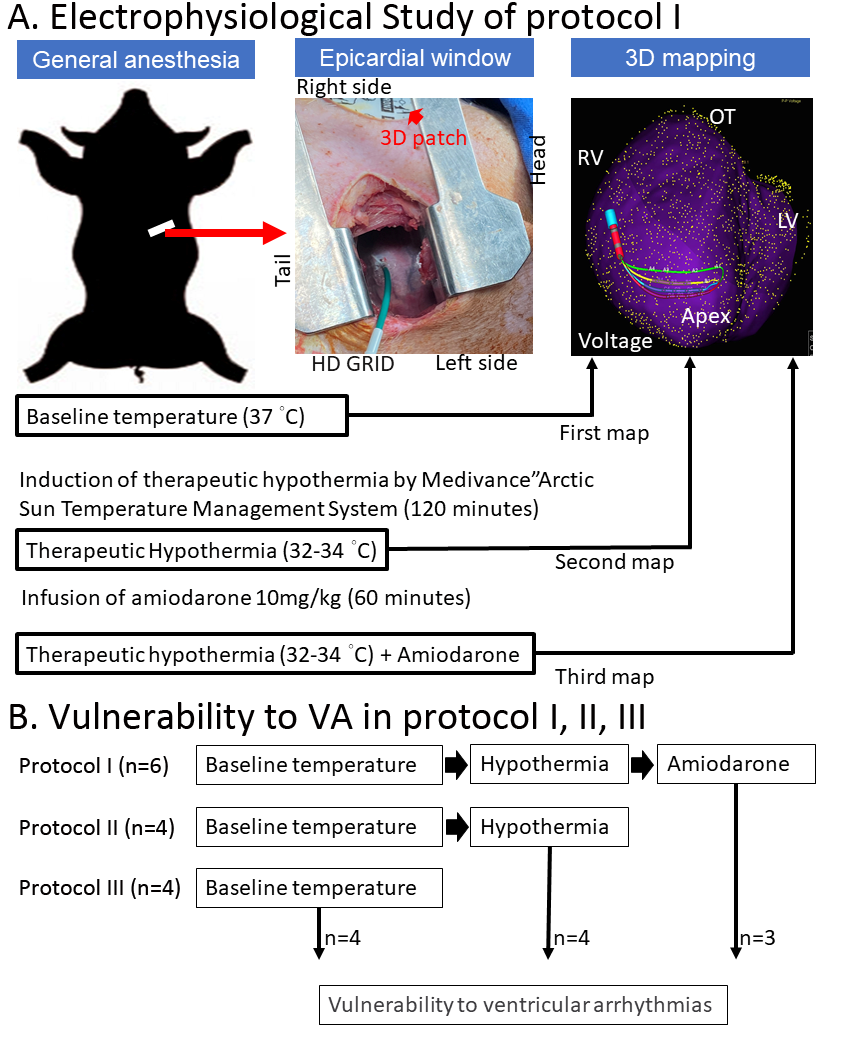
**

(A) In the first part of protocol I, epicardial window was created for the high-density epicardial mapping under sinus rhythm and right ventricular pacing rhythm by using multi-electrode mapping catheter in 6 pigs. Epicardial mapping was repeated after the induction of TH and after the infusion of amiodarone.

(B) In the second part of protocol I, vulnerability to VA was performed in 3 swine. Another 4 and 4 swine underwent protocol II and protocol III respectively. In the protocol II and III, vulnerability to VA was examined under TH and BT respectively without 3D mapping /electrophysiological study. In the protocol II, induction of TH was performed and vulnerability to VA was conducted in 4 swine after an observation time of 60 minutes. In the protocol III, vulnerability to VA was conducted in 4 swine after an observation time of 120 minutes.

OT = outflow tract; RV = right ventricle; LV = left ventricle; 3D = three-dimensional; VA = ventricular arrhythmias

**Supplemental Figure 2: Segmentation of the epicardium**


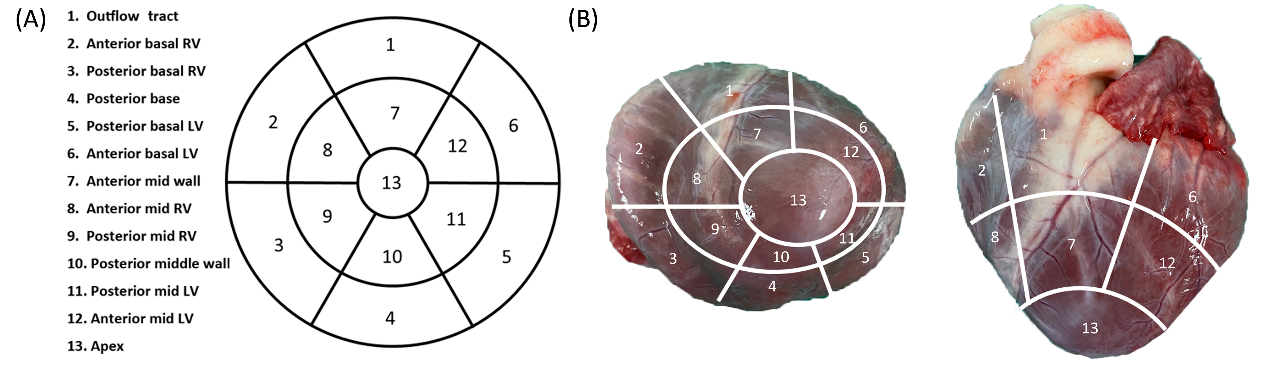


The circumferential locations in the base and mid-cavity are anterior, RV anterior, RV posterior, posterior, and LV posterior. (A) Thirteen segments were assigned according to the modified AHA segmentation. (B) The example of the segmentation in the epicardial ventricle from inferior view (left panel) and anterior view (right panel).

**Supplemental Figure 3. Bipolar epicardial voltage after therapeutic hypothermia or the treatment of amiodarone**


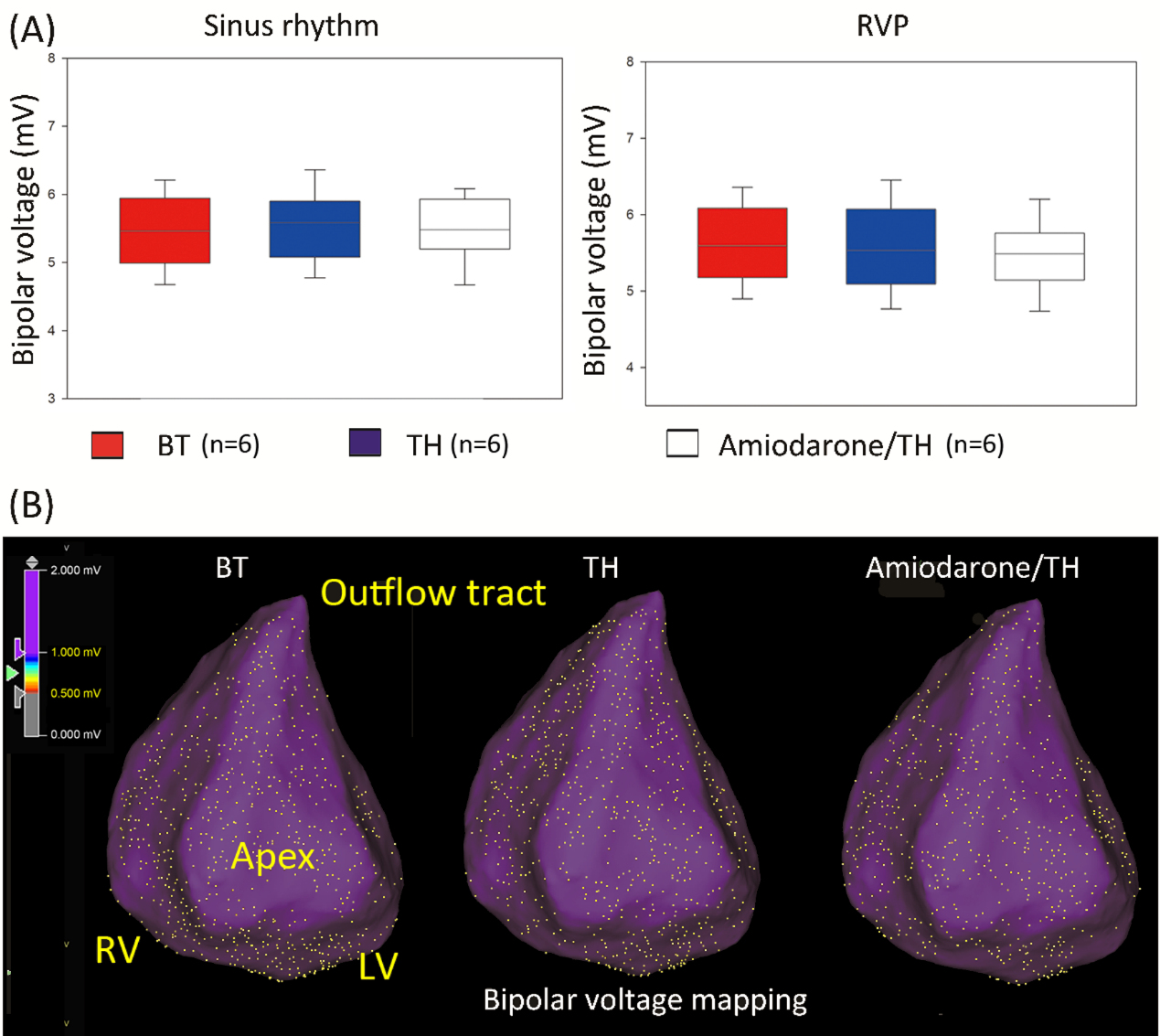


A: The mean bipolar voltage was similar after the induction of TH and after the infusion of amiodarone despite the rhythm.

B: The three-dimensional electro-anatomic mapping demonstrated the bipolar voltage mapping during baseline temperature, after TH, and after the infusion of amiodarone. The Panel B showed a left anterior-oblique (LAO) view to the heart. The upper part indicated anterior aspect of the heart and the lower part indicated the posterior aspect of the heart. From a LAO view, the LV was in the right side and RV in the left side. The landmark was highlighted in yellow color. The purple color indicated the voltage more than 1.0 mV, which was within normal range.

BT: baseline temperature; HT = therapeutic hypothermia; SR: sinus rhythm;

RVP: right ventricular pacing

**Supplemental Figure 4: Activation pattern of the ventricular epicardium**


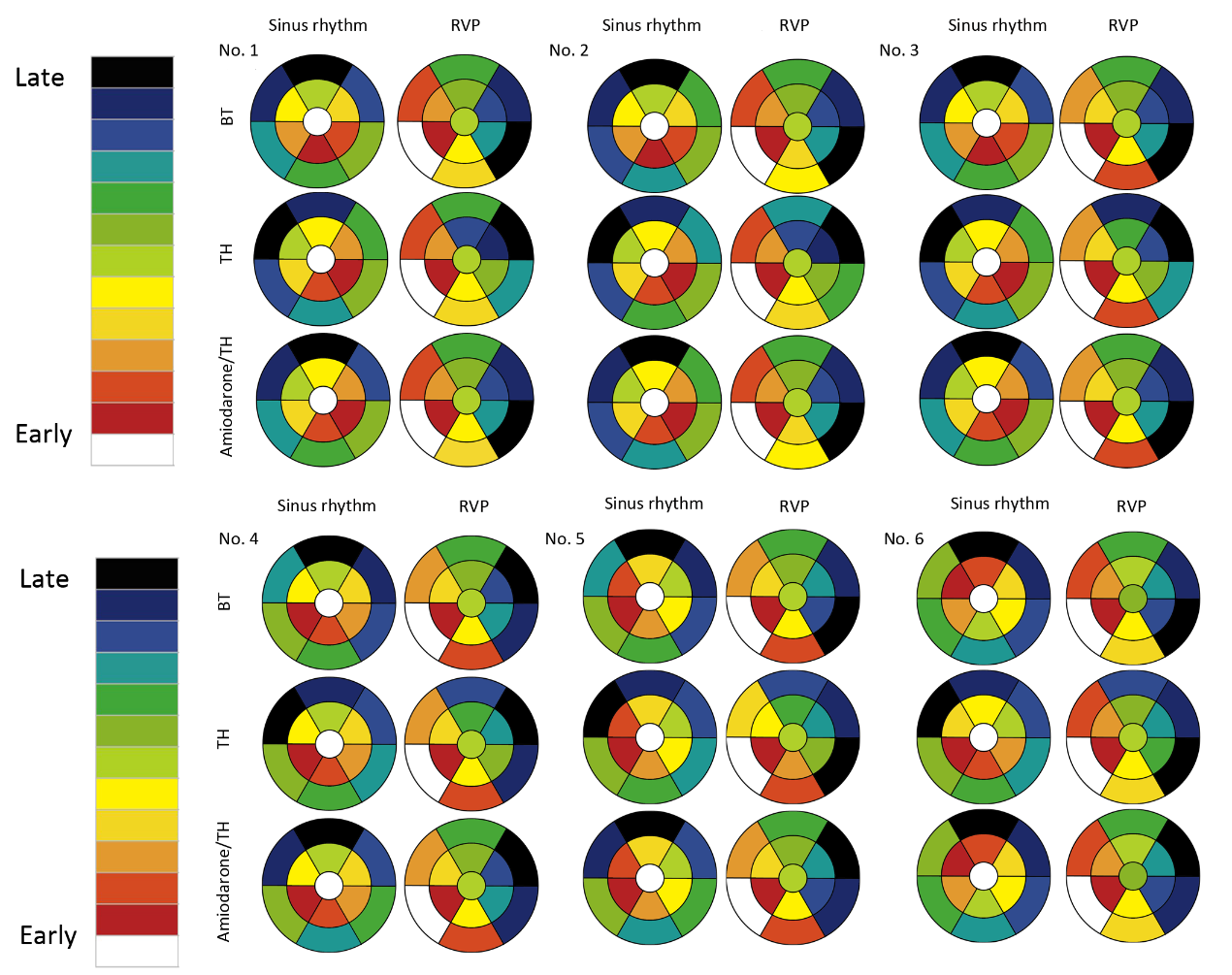


The activation pattern of the ventricular epicardium during SR and RV pacing rhythm at baseline, TH, and after the infusion of amiodarone.

BT: baseline temperature; TH = therapeutic hypothermia; SR: sinus rhythm;

RVP: right ventricular pacing

**Supplemental Figure 5: Western blot of the connexin 43 between control group and study group.**


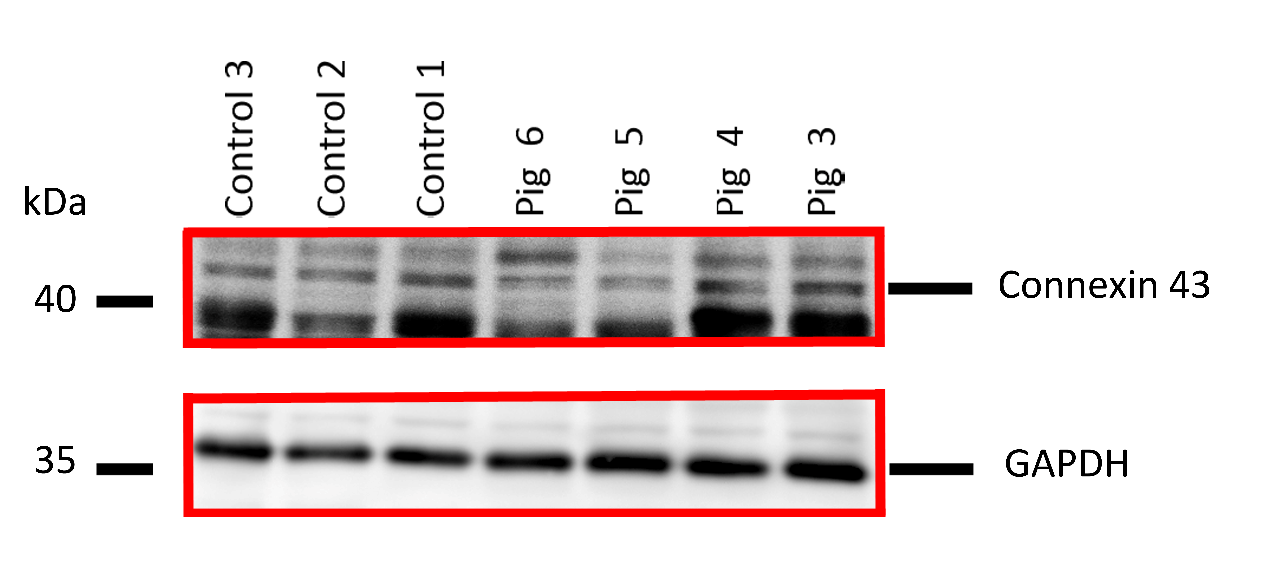


The images was gathered from different locations of the same gel and time.

The expression of connexin 43 was similar between control group without TH and the study group underwent TH. There was no significant difference between these two groups.

GAPDH =Glyceraldehyde-3-Phosphate Dehydrogenase; TH = therapeutic hypothermia

Reference

1. Wass CT, White RD, Schroeder DR, Mirzoyev SA, Warfield KT. Therapeutic hypothermia for out-of-hospital ventricular fibrillation survivors: a feasibility study comparing time to achieve target core temperature using conventional conductive cooling versus combined conductive plus pericranial convective cooling. *J Cardiothorac Vasc Anesth*. 2013;27:288-291. doi: 10.1053/j.jvca.2012.11.026

2. Weng CH, Chung FP, Chen YC, Lin SF, Huang PH, Kuo TB, Hsu WH, Su WC, Sung YL, Lin YJ, et al. Pleiotropic Effects of Myocardial MMP-9 Inhibition to Prevent Ventricular Arrhythmia. *Sci Rep*. 2016;6:38894. doi: 10.1038/srep38894
